# Supplementary material for: A Critical View of the Application of the APEX Software (Aqueous Photochemistry of Environmentally-Occurring Xenobiotics) to Predict Photoreaction Kinetics in Surface Freshwaters
Source: Molecules. 2019 Dec 18;25(1):9. doi: 10.3390/molecules25010009 (PMC7017383; doi:10.3390/molecules25010009)
Supplement: Supplementary file 1 [file molecules-25-00009-s001.zip › 3.2.4_gcc-4.4.0.zip!/3.2.4_gcc-4.4.0/3.2.4_gcc-4.4.0/doc/octave/html/interpreter/Contour-groups.html]

Contour groups - Untitled


Next: Error bar series,
Previous: Bar series,
Up: Object Groups


---

##### 15.2.8.4 Contour groups

Contour group objects are created by the `contour`, `contourf`
and `contour3` functions. The are equally one of the handles returned
by the `surfc` and `meshc` functions. The properties of the contour
group are

`contourmatrix`: A read only property that contains the data return by `contourc` used to create the contours of the plot. `fill`: A radio property that can have the values "on" or "off" that flags whether the contours to plot are to be filled. `zlevelmode` `zlevel`: The radio property `zlevelmode` can have the values "none", "auto" or "manual". When its value is "none" there is no z component to the plotted contours. When its value is "auto" the z value of the plotted contours is at the same value as the contour itself. If the value is "manual", then the z value at which to plot the contour is determined by the `zlevel` property. `levellistmode` `levellist` `levelstepmode` `levelstep`: If `levellistmode` is "manual", then the levels at which to plot the contours is determined by `levellist`. If `levellistmode` is set to "auto", then the distance between contours is determined by `levelstep`. If both `levellistmode` and `levelstepmode` are set to "auto", then there are assumed to be 10 equal spaced contours. `textlistmode` `textlist` `textstepmode` `textstep`: If `textlistmode` is "manual", then the labelled contours is determined by `textlist`. If `textlistmode` is set to "auto", then the distance between labelled contours is determined by `textstep`. If both `textlistmode` and `textstepmode` are set to "auto", then there are assumed to be 10 equal spaced labelled contours. `showtext`: Flag whether the contour labels are shown or not. `labelspacing`: The distance between labels on a single contour in points. `linewidth` `linestyle` `linecolor`: The properties of the contour lines. The properties `linewidth` and `linestyle` are similar to the corresponding properties for lines. The property `linecolor` is a color property (see Colors), that can also have the values of "none" or "auto". If `linecolor` is "none", then no contour line is drawn. If `linecolor` is "auto" then the line color is determined by the colormap. `xdata` `ydata` `zdata`: The original x, y, and z data of the contour lines. `xdatasource` `ydatasource` `zdatasource`: Data source variables.
